# Neonatal outcomes in preterm infants with severe congenital heart disease: a national cohort analysis

**DOI:** 10.3389/fped.2024.1326804

**Published:** 2024-04-25

**Authors:** Safwat Aly, Ibrahim Qattea, Mohammad O. Kattea, Hany Z. Aly

**Affiliations:** ^1^ Department of Cardiology, Boston Children’s Hospital, Boston, MA, United States; ^2^ Department of Pediatrics, Harvard Medical School, Boston, MA, United States; ^3^ Department of Pediatrics, Nassau University Medical Center, East Meadow, NY, United States; ^4^ Department of Neonatology, Cleveland Clinic, Cleveland, OH, United States

**Keywords:** prematurity, gestational age, congenital heart disease, outcomes, necrotizing enterocolitis

## Abstract

**Background:**

Prematurity and congenital heart disease (CHD) are the leading causes of neonatal mortality and morbidity. Limited data are available about the outcomes of premature infants with severe CHD.

**Methods:**

We queried The National Inpatient Database using ICD-10 codes for premature patients (<37 weeks) with severe CHD from 2016 to 2020. Severe CHDs were grouped into three categories: A. left-sided lesions with impaired systemic output, B. Cyanotic CHD, and C. Shunt lesions with pulmonary overcirculation. Patients with isolated atrial or ventricular septal defects and patent ductus arteriosus were excluded. We also excluded patients with chromosomal abnormalities and major congenital anomalies. Patients' demographics, clinical characteristics, and outcomes were evaluated by comparing premature infants with vs. without CHD adjusting for gestational age (GA), birth weight, and gender.

**Results:**

A total of 27710 (1.5%) out of 1,798,245 premature infants had severe CHD. This included 27%, 58%, and 15% in groups A, B, and C respectively. The incidence of severe CHD was highest between 25 and 28 weeks of gestation and decreased significantly with increasing GA up to 36 weeks (*p* < 0.001). Premature infants with severe CHD had a significantly higher incidence of neonatal morbidities including necrotizing enterocolitis (NEC) [OR = 4.88 (4.51–5.27)], interventricular hemorrhage [OR = 6.22 (5.57–6.95)], periventricular leukomalacia [OR = 3.21 (2.84–3.64)] and bronchopulmonary dysplasia [OR = 8.26 (7.50–10.06) compared to preterm infants of similar GA without CHD. Shunt lesions had the highest incidence of NEC (8.5%) compared to 5.3% in cyanotic CHD and 3.7% in left-sided lesions (*p* < 0.001). Mortality was significantly higher in premature infants with CHD compared to control [11.6% vs. 2.5%, *p* < 0.001]. Shunt lesions had significantly higher mortality (11.0%) compared to those with left-sided lesions (8.3%) and cyanotic CHD (6.4%), *p* < 0.001.

**Conclusion:**

Premature infants with severe CHD are at high risk of neonatal morbidity and mortality. Morbidity remains increased across all GA groups and in all CHD categories. This significant risk of adverse outcomes is important to acknowledge when managing this patient population and when counseling their families. Future research is needed to examine the impact of specific rather than categorized congenital heart defects on neonatal outcomes.

## Introduction

Prematurity continues to be the leading cause of neonatal mortality in developed countries (1). The association between gestational age (GA) and neonatal mortality is well known. Gestational age is the major predictor of mortality in extremely preterm infants without congenital anomalies. Even those born at 34–36 weeks of gestation have a higher mortality risk than full-term infants (1, 2). Most of the studies that examined morbidity and mortality in preterm infants excluded congenital heart disease (CHD) to better distinguish the attribute of prematurity on neonatal death (3).

Congenital heart disease is the most common birth defect, affecting −40,000 infants per year in the United States representing −0.8% of all births with −0.2% of them reported to have critical CHD (4, 5). Previous reports demonstrated that CHDs are more frequently encountered in preterm than full-term neonates (6, 7). Having a congenital heart defect raises the risk of premature birth by 2-to-3 fold compared to neonates without CHD (6–8). It is well-known that severe CHDs are associated with higher mortality rates in full-term infants (6–9).

Despite the significant association between prematurity and CHD, less is known about the impact of GA on outcomes in patients with severe CHD. The few studies that examined the outcomes of premature infants with CHD carried significant limitations including relatively small numbers of premature infants, older birth cohorts (before 2005), excluded extreme preterm or moderate/late preterm infants, or did not account for important confounders such as GA, birth weight (BW), and gender (1, 3, 6–9). Moreover, the inclusion of mixed forms of CHDs including minor ones, and the substantial number of patients with non-cardiac anomalies and genetic syndromes might have contributed to the significant variations in the rates of adverse outcomes reported (3, 10, 11).

Therefore, in the current study, we aimed to use the largest national database to (1) Examine the frequency of severe CHD in premature infants born at various GAs, and (2) Quantify the effect of GA on neonatal mortality and major neonatal morbidities in preterm infants with different categories of severe CHD by comparing them to a control group of preterm infants without any CHD.

## Methods

### Data source

This observational study used deidentified patient data from the National Inpatient Sample (NIS) database during the period from January 1, 2016 to December 31, 2020. This database is part of the Healthcare Cost and Utilization Project (HCUP) and has been used for many previous projects. The NIS database contains −8 million hospital stays each year and randomly samples 20% of the discharges from the participating hospitals and is weighed to accurately reflect 100% of all US hospital discharges (12). Procedure and diagnostic codes are documented using the International Classification of Diseases. In the current study, we only used International Classification of Disease, Tenth Revisions, and Clinical Modification (ICD10-CM) to identify our patient population.

### Patient selection

The study was granted an exempt status from the Institutional Review Board given the use of a deidentified and publicly available database. Patients were included in the study if they were born prematurely (<37 0/7 weeks of gestation) with severe CHD. Severe CHDs were categorized into three main groups based on the underlying physiology: *Category A*: left-sided lesions with impaired systemic output, *Category B*: cyanotic CHD with persistent cyanosis, and *Category C*: Shunt lesions with pulmonary over-circulation and subsequent congestive heart failure. Patients were excluded from the study if they had only a simple or minor CHD including an isolated atrial septal defect, ventricular septal defect, or patent ductus arteriosus. The study excluded patients with genetic or chromosomal abnormalities and/or major congenital anomalies. Patients who were transferred between hospitals were only included at the receiving hospital to avoid duplication. The list of all CHDs included in the study and their respective ICD-10 codes is shown in Supplementary Table S1.

### Identification of variables

The NIS database was queried for patients’ demographics and clinical characteristics. The main outcomes of interest included in-hospital mortality and major neonatal morbidities. Non-cardiac neonatal morbidities included necrotizing enterocolitis [ICD-10 codes: P77.1, P77.2, and P77.3], severe interventricular hemorrhage (greater than grade II) [P52.2 and P52.0], periventricular leukomalacia (PVL) [P91.2], and bronchopulmonary dysplasia (BPD) [P27.1].

Patient characteristics and outcomes were evaluated by comparing premature infants with severe CHD to a control group of premature infants with matching GA but without any CHD. We also examined the frequency of different procedures (extracorporeal membrane oxygenation support, tracheostomy, and gastrostomy) between the two groups and their association with outcomes.

### Statistical analysis

Data were presented using the mean and standard deviation for parametric continuous variables, the median and interquartile range for nonparametric continuous variables, and the frequencies and proportions for categorical variables. To examine the occurrence of CHD and its association with outcomes of interest, we classified premature infants based on GA into the following categories; extremely premature [≤27 6/7 weeks], very premature [28 0/7 to 31 6/7 weeks], moderate preterm [32 0/7 to 33 6/7 weeks], late preterm [34 0/7 to 36 6/7 weeks]. Premature infants with severe CHD were compared to those without any CHD using the Mann–Whitney *U*-test for continuous characteristics and chi-square tests for categorical characteristics. Both cases and controls were matched for GA, gender, and BW. Patients with CHD were further grouped into three main categories (left-sided CHD, cyanotic CHD, and shunt lesions with pulmonary overcirculation). The odds ratios (OR) of developing the different neonatal outcomes of interest in the different CHD categories in comparison to controls were evaluated using chi-square tests. *P* < 0.05 was considered significant. Data analysis was performed using SAS version 9.4 (SAS Institute Inc., Cary, NC).

## Results

Between 2016 and 2020, there were 1,827,044 preterm births in the NIS database. This included 27,710 (1.5%) with severe CHD. Premature infants with severe CHD were classified based on the underlying cardiovascular physiology into three main groups including; −26% left-sided lesions with impaired systemic output (*category A*), −62% with sustained cyanosis (*category B*), and −12% with shunt lesions and pulmonary overcirculation (*category C*).

**Figure 1:**
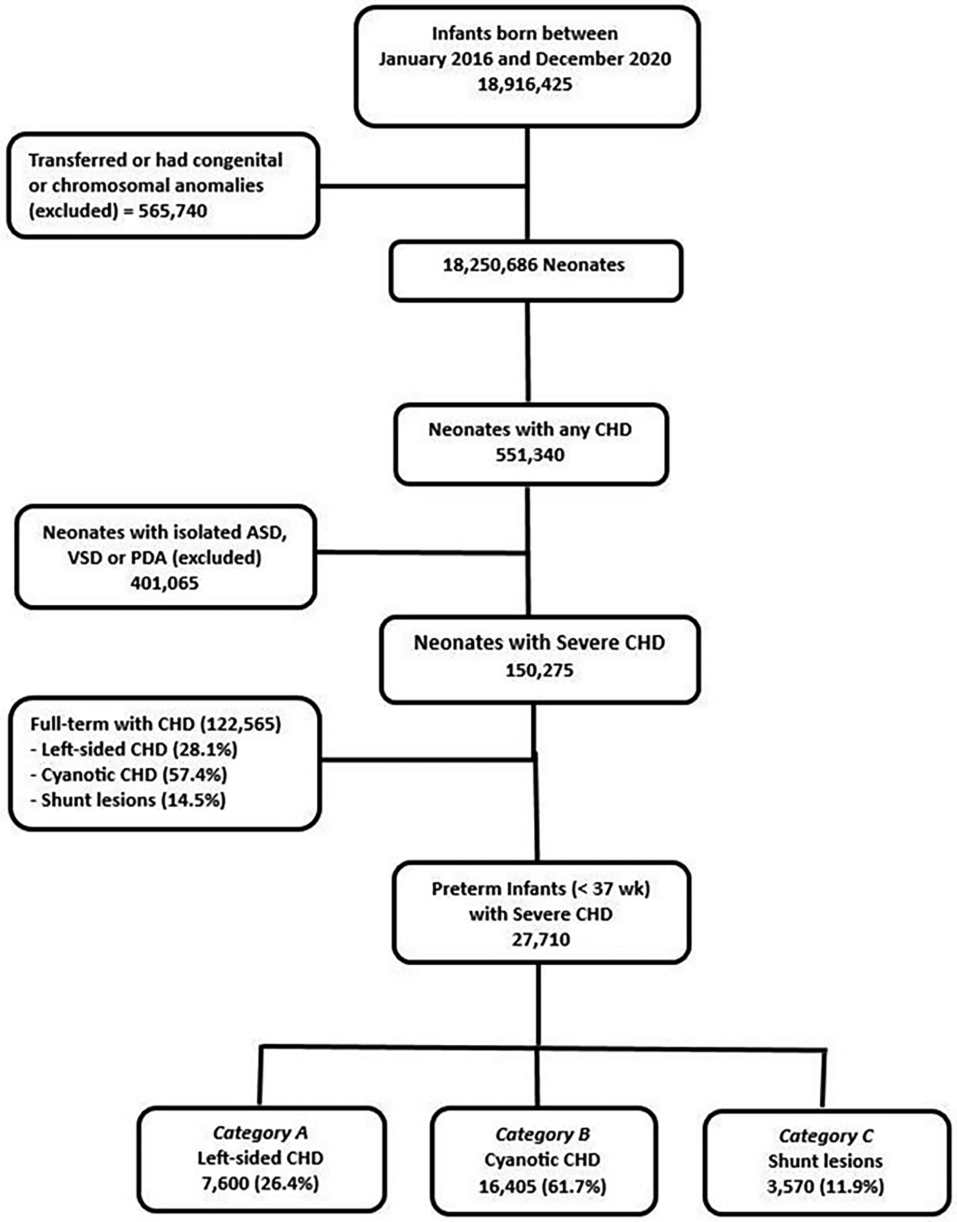
Flow chart for the patient population.

There was no significant difference in the distribution of the three different categories of CHD in preterm infants when compared to full-term infants born with similar CHDs during the study period. Figure 1 illustrates the flow chart for the study population.

The incidence of severe CHD in premature birth (premature patients with severe CHD/all preterm births in the same year) varied from 1.39% to 1.65% per year. The association between prematurity and incidence of severe CHD was more frequent in males (54%) and whites (41%). Low median household income was associated with a higher frequency of having a premature infant with severe CHD. Regional differences existed in the association of prematurity and CHD. Demographics of premature infants with vs. without severe CHD are demonstrated in Table 1.

**Table 1:** Demographics and distribution of the study population.

		Preterm infants with severe CHD		Preterm infants without severe CHD		*P*-value
Indicator of sex	Male	15,025	54.2%	960,340	53.4%	
	Female	12,685	45.8%	837,905	46.6%	<0.001
Race (uniform)	White	11,395	41.1%	776,560	43.2%	<0.001
	Black	5,110	18.4%	333,740	18.6%	
	Others	11,205	40.4%	689,035	38.3%	
Primary expected payer (uniform)	Medicare/Medicaid	15,320	55.3%	927,380	51.6%	<0.001
	Private insurance	10,565	38.1%	740,725	41.2%	
	Others	1,825	6.6%	131,230	7.3%	
Calendar year	2016	5,335	19.3%	358,380	19.9%	<0.001
	2017	5,125	18.5%	361,335	20.1%	
	2018	5,620	20.3%	361,865	20.1%	
	2019	5,730	20.7%	366,545	20.4%	
	2020	5,900	21.3%	351,210	19.5%	
Median household income national quartile for patient ZIP Code	0–25th percentile	9,290	33.5%	562,315	31.3%	<0.001
	26th–50th percentile	7,310	26.4%	454,390	25.3%	
	51st–75th percentile	5,975	21.6%	419,545	23.3%	
	76th–100th percentile	4,860	17.5%	345,755	19.2%	
Location/teaching status of the hospital	Rural	155	0.6%	61,330	3.4%	<0.001
	Urban nonteaching	1,360	4.9%	183,055	10.2%	
	Urban teaching	14,570	52.6%	837,485	46.6%	
Region of hospital	Northeast	2,110	7.6%	158,395	8.8%	<0.001
	Midwest	3,490	12.6%	239,510	13.3%	
	South	7,015	25.3%	450,180	25.0%	
	West	3,470	12.5%	233,785	13.0%	
Control/ownership of hospital	Government, nonfederal	2,270	8.2%	128,090	7.1%	<0.001
	Private, non-profit	12,220	44.1%	817,030	45.4%	
	Private, invest-own	1,595	5.8%	136,750	7.6%	

**Figure 2:**
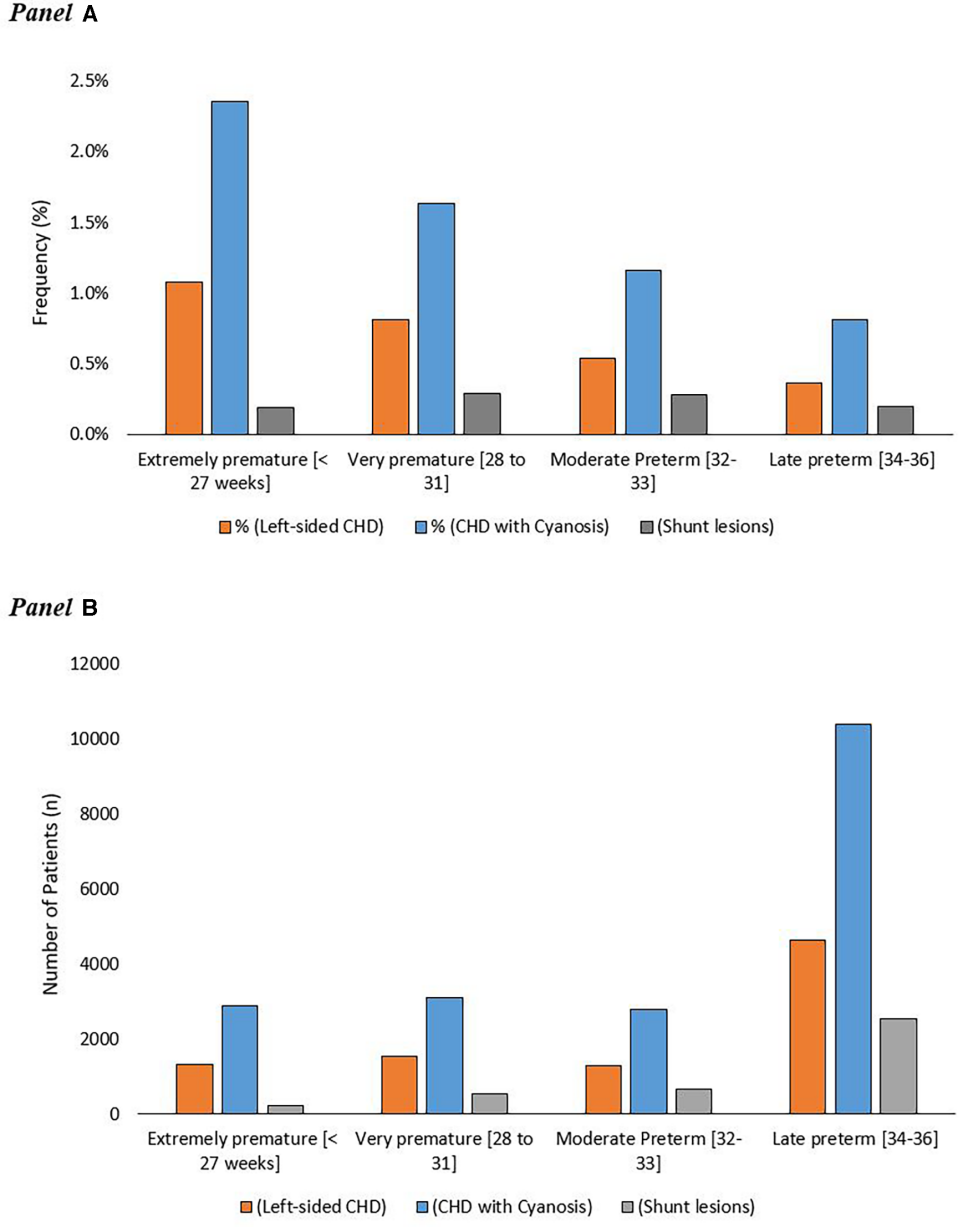
Frequency of different categories of severe congenital heart disease in premature infants in relation to gestational ages (**A**) the absolute number of premature patients with different categories of severe CHD in relation to their gestational age (**B**).

A total of 3.2% of extremely premature infants (≤27 weeks) had severe CHD compared to 2.3% in very preterm, 1.5% in moderate preterm, and 1.1% in late preterm infants (*p* < 0.01). The numbers and frequencies of the different categories of severe CHD in preterm infants according to their GA category are demonstrated in Figure 2.

Premature birth of infants with severe CHD was more likely to occur in pregnancies complicated by maternal hypertension [3.9% vs. 0.6%, *p* < 0.001, OR of 3.19 (2.99–3.41)]. Preexisting diabetes and gestational diabetes were associated with a higher frequency of severe CHD in preterm infants [10.1% vs. 1.2%, *p* < 0.001, OR of 7.86 (7.54–8.19) and 5.8% vs. 2.0%, *p* < 0.001, OR of 3.02 (2.87–3.18), respectively], Tables 2, 3.

**Table 2:** Clinical outcomes of study population: *cases* (preterm infants with severe CHD) versus *controls* (preterm infants without any CHD).

		Preterm with severe CHD		Preterm without severe CHD		*P*-value
Maternal factors						
Maternal hypertension	No	26,640	96.1%	18,780,255	99.4%	
	Yes	1,070	3.9%	114,510	0.6%	<0.001
Gestational diabetes	No	26,110	94.2%	18,510,576	98.0%	
	Yes	1,600	5.8%	384,190	2.0%	<0.001
Preexisting diabetes	No	24,915	89.9%	18,659,036	98.8%	
	Yes	2,795	10.1%	235,730	1.2%	<0.001
NIS	No	27,325	98.6%	18,748,466	99.2%	
	Yes	385	1.4%	146,300	0.8%	<0.001
CS	No	16,860	60.8%	13,398,563	70.9%	
	Yes	10,850	39.2%	5,496,202	29.1%	<0.001
Neonatal morbidities						
Necrotizing enterocolitis	No	26,625	96.08%	1,782,189	99.05%	
	Yes	1,085	3.92%	17,145	0.95%	<0.001
Severe IVH	No	27,250	98.30%	1,819,939	99.60%	
	Yes	460	1.70%	6,805	0.40%	<0.001
Periventricular leukomalacia	No	27,320	98.60%	1,819,589	99.60%	
	Yes	390	1.40%	7,155	0.40%	<0.001
Bronchopulmonary dysplasia	No	25,295	91.30%	1,785,014	97.70%	
	Yes	2,415	8.70%	41,730	2.30%	<0.001
Arrhythmia	No	26,885	97.02%	1,795,889	99.81%	
	Yes	825	2.98%	3,445	0.19%	<0.001
Pulmonary hypertension	No	27,415	98.94%	1,798,079	99.93%	
	Yes	295	1.06%	1,255	0.07%	<0.001
Sepsis	No	27,070	97.69%	1,792,644	99.63%	
	Yes	640	2.31%	6,690	0.37%	<0.001
Acute renal failure	No	26,085	94.14%	1,789,069	99.43%	
	Yes	1,625	5.86%	10,265	0.57%	<0.001
Pleural effusion	No	27,530	99.35%	1,798,829	99.97%	
	Yes	180	0.65%	505	0.03%	<0.001
Pneumothorax	No	27,595	99.58%	1,798,274	99.94%	
	Yes	115	0.42%	1,060	0.06%	<0.001
Cardiac arrest	No	27,635	99.73%	1,799,119	99.99%	
	Yes	75	0.27%	215	0.01%	<0.001
ECMO	No	27,360	98.74%	1,798,899	99.98%	
	Yes	350	1.26%	435	0.02%	<0.001
Tracheostomy	No	27,630	99.71%	1,799,009	99.98%	
	Yes	80	0.29%	325	0.02%	<0.001
Gastrostomy	No	27,665	99.84%	1,799,164	99.99%	
	Yes	45	0.16%	170	0.01%	<0.001
Catheterization	No	27,635	99.73%	1,799,329	99.99%	
	Yes	75	0.27%	15	0.01%	<0.001
Died during hospitalization	No	24,480	88.30%	1,779,409	97.40%	
	Yes	3,210	11.60%	46,290	2.50%	<0.001
Length of stay for alive		42 (26–81)		17 (9–22)		
Length of stay for died		25 (18–32)		9 (6–17)		<0.001

Both cases and controls were matched for GA, gender, and birth weight.

**Table 3:** The odds ratio for the association of maternal characteristics, neonatal comorbidities, and need for interventions in preterm infants with severe CHD.

	*P*-value	Odds-ratio (confidence interval)
Maternal hypertension	<0.001	3.19 (2.99–3.41)
Gestational diabetes	<0.001	3.02 (2.87–3.18)
Preexisting diabetes	<0.001	7.86 (7.54–8.19)
Cesarean section	<0.001	1.41 (1.37–1.44)
Neonatal abstinence syndrome	<0.001	1.7 (1.53–1.89)
Necrotizing enterocolitis	<0.001	4.88 (4.51–5.27)
Severe interventricular hemorrhage	<0.001	6.22 (5.57–6.95)
Periventricular leukomalacia	<0.001	3.21 (2.84–3.64)
Bronchopulmonary dysplasia	<0.001	18.26 (14.50–20.06)
Arrhythmia	<0.001	9.69 (8.86–10.61)
Pulmonary hypertension	<0.001	5.62 (4.83–6.54)
Heart failure	<0.001	5.91 (4.98–7.03)
Sepsis	<0.001	2.76 (2.49–3.06)
Acute kidney injury	<0.001	6.66 (6.19–7.16)
Pleural effusion	<0.001	4.84 (3.96–5.9)
Pneumothorax	<0.001	2.99 (2.35–3.8)
Cardiac arrest	<0.001	1.57 (1.15–2.16)
Extracorporeal membrane oxygenation	<0.001	1.98 (1.71–2.3)
Gastrostomy	<0.001	8.49 (5.6–12.87)
Gastrostomy	<0.001	8.49 (5.6–12.87)
Tracheostomy	<0.001	3.06 (2.33–4.02)

The frequency of being born before 25 weeks of gestation with severe CHD was much less compared to the other gestational weeks till the 34th week. The highest frequency of premature birth associated with severe CHD occurred in premature infants born after 25 weeks and before 28 weeks of gestation. After the 28th weeks of gestation, there was a steady decrease in the incidence of severe CHD in premature infants until reaching the 36th week of gestation. Figure 2 and Supplementary Figure S1.

**Figure 3:**
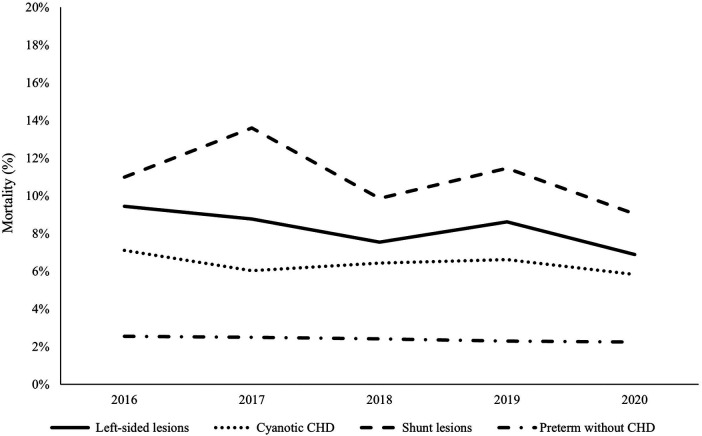
Mortality trends in premature infants with congenital heart disease versus controls during the study period (2016–2020). The dashed line represents shunt lesions, the solid line represents left-sided lesions, the dotted line represents cyanotic CHDs, and the dashed-dotted line represents the control group (preterm infants without CHD). Shunt lesions were associated with the highest mortality rates while cyanotic CHDs were associated with the lowest mortality. There was no significant improvement in survival of preterm infants with CHD during the study period.

In-hospital mortality was significantly higher in premature infants with CHD compared to those without CHD [11.6% vs. 2.5%, *p* < 0.001]. Shunt lesions with pulmonary over-circulation had significantly higher mortality (11.0%) compared to left-sided lesions (8.3%) and cyanotic CHD (6.4%), *p* < 0.001. There was no statistically significant difference in the trends of mortality within each CHD category over the study period. Trends of in-hospital mortality in premature infants with the different categories of CHD compared to controls are shown in Figure 3.

**Figure 4:**
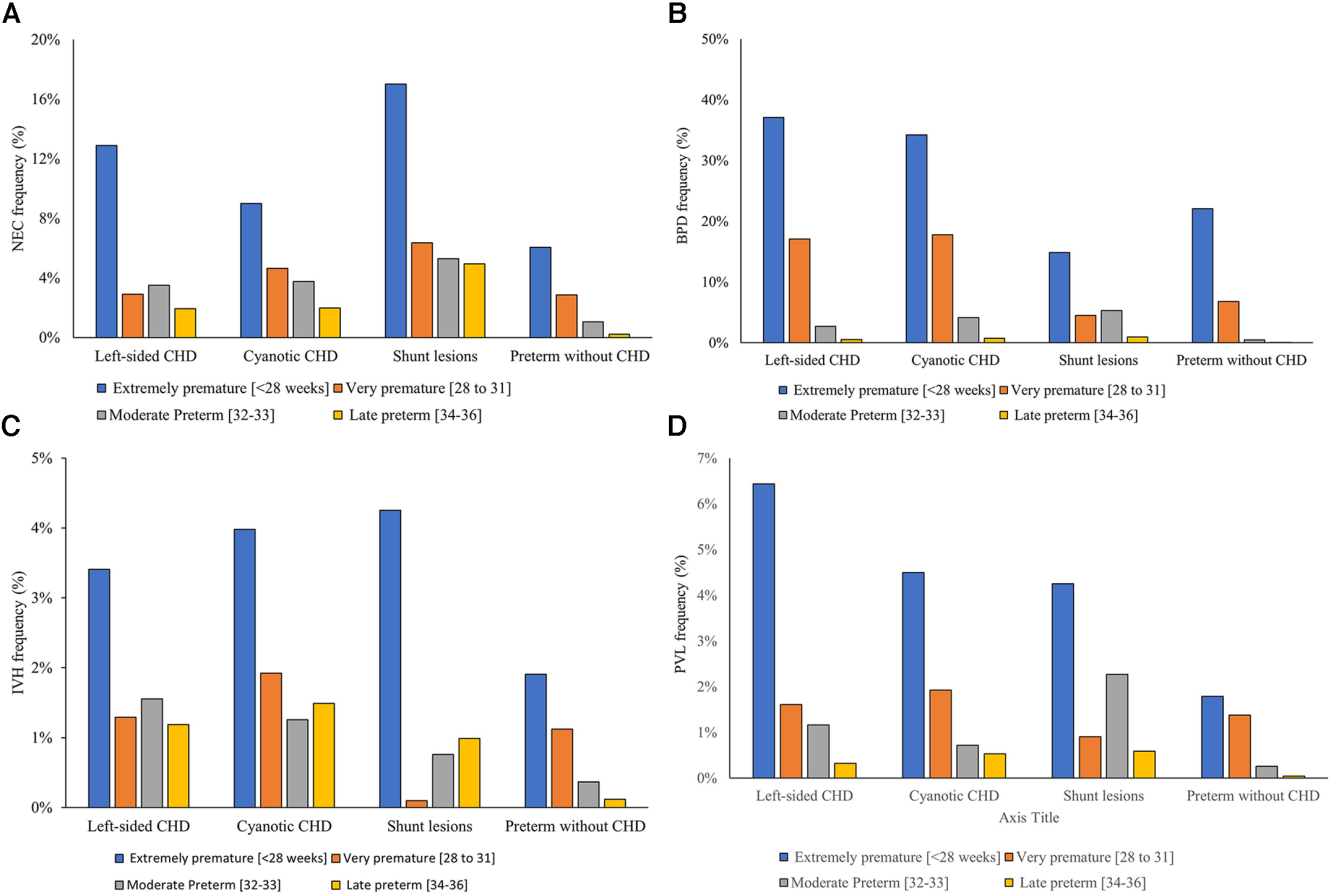
Gestational age-specific major neonatal morbidity rates in infants with versus without severe congenital heart disease. (**A**) Necrotizing enterocolitis, (**B**) bronchopulmonary dysplasia, (**C**) interventricular hemorrhage, (**D**) periventricular leukomalacia.

Major neonatal morbidities were more frequent in premature infants with CHD compared to controls. Necrotizing enterocolitis was more common in premature infants with CHD [3.92% vs. 0.95%, *P* < 0.001, 4.88 (4.51–5.27)]. Extremely preterm infants (born ≤27 weeks) with severe CHD were associated with the highest frequency of having NEC (−11%) compared to −4% for those born between 28 and 32 weeks of gestation and −2% for late preterm infants with CHD. Shunt lesions were associated with the highest incidence of NEC in preterm infants occurring in 8.5% compared to 5.3% in the cyanotic CHD group and 3.7% in left-sided CHD Figure 4A.

Similarly, bronchopulmonary dysplasia rates were significantly higher in premature infants with CHD compared to those without CHD [18.7% vs. 2.3%, *p* < 0.001, OR = 18.3 (14.5–20.1)]. The frequency of developing BPD was significantly higher in patients with cyanotic CHD and left-sided CHD (12.6% and 11.8%, respectively) compared to 6.2% in those with shunt lesions (*p* < 0.001) Figure 4B.

Premature infants with CHD had a higher frequency of having severe neonatal brain injury (defined as having IVH grades III or IV (1.7% vs. 0.4%, *p* < 0.001, 6.2 (5.6–6.9) or PVL [1.4% vs. 0.4%, *p* < 0.001, 3.2 (2.8–3.6)]. Figures 4C,D. Cyanotic CHD had a significantly higher incidence of severe IVH (4.7%) compared to left-sided lesions (3.8%) and shunt lesions (3.6%), *p* < 0.001 Figure 4 illustrates the frequency of the different neonatal morbidities associated with the different categories of severe CHD in relation to the degree of prematurity.

Premature infants with CHD had a higher frequency of sepsis (2.3% vs. 0.3%, *p* < 0.001) and acute renal failure (5.8% vs. 0.5%, *p* < 0.001) compared to those without CHD. Premature infants with CHD received more non-cardiac procedures compared to controls including tracheostomy and gastrostomy tubes Table 4.

**Figure 5:**
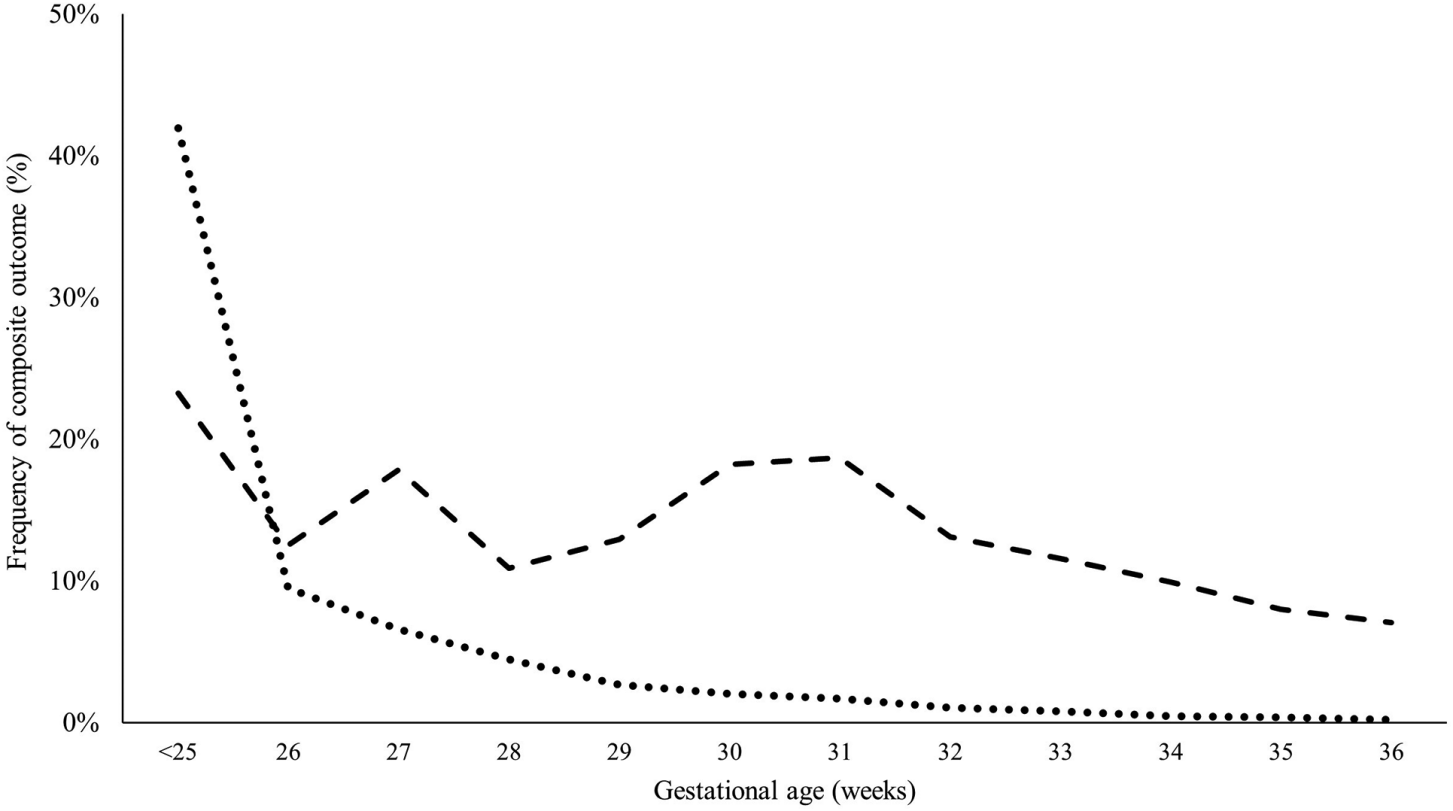
Probability of having composite outcome (mortality or severe morbidity) in infants with significant CHD versus controls based on gestational age. Adjusted for birth weight, sex, and multiple gestation.

Premature infants with CHD had higher odds of having the composite outcome (mortality and major neonatal morbidity) compared to those without CHD after adjusting for GA, BW, and gender (27.3% % vs. 6.6%, *p* < 0.001). Extremely preterm infants without CHD had a higher frequency of developing the composite outcome compared to those with similar GA but without CHD (47.6% vs. 35.9%, *p* < 0.001). Similarly, very preterm, and moderate preterm had a higher frequency of developing the composite outcome compared to those with similar GA but without CHD (25.2% vs. 18.6%, *p* < 0.001 and 12.0% vs. 5.1%, *p* < 0.001, respectively). The occurrence of composite outcomes continued to be significantly higher in late preterm infants compared to controls (9.6% vs. 4.2%, *p* < 0.001). Figure 5 illustrates the differences in developing the composite outcome between the two groups (cases vs. controls) in relation to their GA.

## Discussion

In the current study, we used the largest national multicenter database to examine the interactions between prematurity and severe CHD over a five-year period. We included 27,710 premature infants with severe CHD and compared them to a control group of premature infants without CHD. The main findings in our study include: (a) premature infants are at higher risk of having severe CHD compared to full-term infants during the study period (1.5% vs. 0.5%, *P* < 0.001 respectively). (b) Severe CHD in premature infants increases the risk of developing non-cardiac prematurity-related complications including NEC, BPD, and severe brain injury. (c) Shunt lesions with pulmonary over-circulation were associated with the highest mortality rates while cyanotic CHDs were associated with the most favorable outcome. (d) Advanced GA was not associated with a significant decline in developing the composite outcome (mortality and major neonatal morbidities) in preterm infants with severe CHD in contrast to those of similar GA but without CHD.

### Prematurity and CHD

The incidence of all CHD in full-term infants is known to be −1% (4–6). However, the true occurrence of CHD especially the severe forms in premature infants is unknown. In the current national analysis of a 5-year period, the incidence of severe CHD in premature infants ranged from 1.39% to 1.65% per year. While full-term infants during the same period had severe CHD ranges of 0.43%–0.49%. This is in agreement with previous reports of high frequency of CHD in preterm infants (6, 7). In an international cohort study, not including the USA, the estimated incidence of severe CHD in premature infants was reported to be −0.8% (3). Despite the international and multicenter nature of the former report, it only included −600 patients with severe CHD. Locally, in a population-based study from the state of California between 2005 and 2012, −0.5% of premature patients included had a critical CHD (13). Our study likely offers more reliable estimates given the enormous patient sample and the wide range of regions included across the United States. Fetal distress occurring secondary to severe CHD might trigger preterm birth in these patients.

***Demographic Variations.*** The association between CHD and prematurity was more frequent in males and in the White population*.* Previous studies reported controversial results regarding the demographic distribution of CHD with preterm birth. In the state of Florida, Nembhard et al. reported higher odds of preterm birth associated with CHD in Black infants compared to the White while the Hispanic population had no increased risk (14).

Desai et al. examined the Kids' Inpatient Database and reported a higher frequency of CHD in the White population (52%) compared to 15% in Black and 19.5% in the Hispanics (15).

***Regional variations*** existed in the association between prematurity and severe CHD. The Northeast had the lowest incidence of CHD in premature infants. This might be, in part, related to the highly diverse nature of the population in this region where dissimilar genes might have played a role in lessening such association. On the other side, the South had the highest incidence of severe CHD in premature infants. The relative unavailability of antenatal screening for CHD and probably the lack of an opportunity to terminate pregnancy might have contributed to higher rates of CHD in preterm infants (16).

In our study, lower socioeconomic status was associated with a higher incidence of severe CHD in premature infants. While the impact of social determinants of health on short and long-term outcomes after the surgical repair of CHD is well-documented, the role of these factors in the development of congenital heart defects is largely unknown, especially in premature infants. However, exposure to toxic environmental agents and environmental pollutants has been linked to prematurity, low BW, and the occurrence of congenital anomalies in multiple previous studies. Exposure to these factors might also contribute to the higher risk of CHD in premature infants (17, 18).

### Mortality

In our study, 11.6% of premature infants with severe CHD died before discharge from the hospital. Significant variations exist in the previous reports of mortality in preterm infants with CHD (19–21). A study of all CHD (including minor or simple CHD) reported the lowest mortality rates (22). While the highest mortality rate of −45% was reported in very low BW infants (<1,500 g, GA 22–29 weeks) (11). Steurer et al. reported a 12.6% in- hospital mortality of infants with critical CHD but this cohort included patients with gestational ages between 22 and 42 weeks (13). An international study reported a mortality rate of 18.6% in preterm infants with severe CHD compared to 8.9% in preterm infants without CHD (3). The high mortality rates even in the control group, compared to −2% in the United States, might reflect not only the variations in CHD outcomes but also the neonatal intensive care of preterm infants in general. Moreover, the inconsistencies in definitions, inclusion criteria, and denominators in these studies might explain the variations in outcomes between studies.

### Necrotizing enterocolitis

The association between NEC and CHD in full-term infants is known (23, 24). It is also known that preterm infants have inherent risk factors for developing NEC (25, 26). However, outcome data about the intersection between prematurity and CHD on the development of NEC is controversial. A report of −600 premature infants with severe CHD did not find an association between NEC and CHD (3). Another study of −200 premature infants with BW <2,500 grams showed a 1.7-fold increase in the risk of NEC compared to all patients admitted to NICU with mixed GAs without adjusting for important variables such as GA and BW (27). Others who studied individual CHDs found a higher risk of NEC in simple CHD as isolated ASD and VSD (28) and an independent association between hypoplastic left heart syndrome or truncus arteriosus and NEC (24). In the current analysis, Preterm infants with severe CHD had a −4-fold increase in the risk of NEC after controlling for GA, BW, and gender. The systemic inflammatory response that occurs in the presence of pulmonary overcirculation and congestive heart failure might elucidate the high risk of NEC in this CHD category. Meanwhile, the impaired systemic output and the subsequent episodic or chronic mesenteric under-perfusion might explain the significant risk of NEC in patients with left-sided obstructive lesions.

### Interventricular hemorrhage

Previous studies reported a wide range of incidence of IVH in infants with CHD ranging from 2 to 24% (13, 29–31). However, these studies were limited to single institutions, small sample sizes, or mostly included full-term infants. The impact of GA on the development of severe IVH in preterm infants with CHD has not been fully examined. Preterm infant literature demonstrated an inverse relationship between the presence and severity of IVH and GA (29, 32). However, this association has not been described in preterm infants with severe CHD. Preterm infants with CHD were reported to experience a wide extent and mostly mild forms of IVH [all the above]. Others reported a lower risk of IVH in preterm infants with CHD compared to controls (9). In the current analysis, the presence of severe CHD in premature infants was associated with a 6-fold increase in the risk of developing severe IVH. Extremely premature infants (<28 weeks) were associated with the highest risk of severe IVH regardless of the category of CHD. It was also noticeable that this risk did not significantly decline with increasing GA. Even late preterm infants (34–36 weeks of gestation) had a significantly higher incidence of severe IVH across all categories of severe CHD compared to controls.

### Periventricular leukomalacia

Full-term infants with severe CHD are at high risk of brain injury and impaired brain maturity. This hit to the brain is “doubled” in premature infants according to previous reports (33, 34). Our study showed a 3-fold increase in the risk of PVL in premature infants with severe CHD in comparison to those without CHD after adjusting for GA, BW, and gender. The combination of brain immaturity, impairment of cerebral blood flow, and low cerebral oxygen saturation might contribute to the high risk of white matter injury and subsequent PVL in premature infants with CHD (34, 35).

### Bronchopulmonary dysplasia

It is not surprising and consistent with previous studies that the incidence of BPD (defined as oxygen dependency at 36 weeks of postmenstrual age) was significantly higher in preterm infants with CHD (3, 36). This high risk might reflect the pathophysiology of the underlying CHD as in cyanotic CHD with an increased need for supplemental oxygen or mechanical ventilation rather than a true BPD.

While shunt lesions with excessive left to right shunt lead to increased pulmonary blood flow, left-sided lesions with impaired forward blood flow might be associated with pulmonary venous hypertension, pulmonary edema, and subsequently impaired lung tissue development and change respiratory mechanics necessitating prolonged mechanical ventilation and oxygen supplementation.

## Strengths and limitations

The current study offers the most contemporary estimation of outcomes in preterm infants (from 22 to 36^+6^ weeks' gestation) with severe CHD based on their gestation ages. We used the NIS which is the largest multicenter database for morbidity and mortality of preterm infants in the USA. To minimize coding errors, we intentionally avoided including ICD-9 codes which were available before 2016. This might have limited our ability to examine the outcomes of premature infants with severe CHD over a longer period. We opted to use ICD-10 codes which provide more detailed information related to cardiac diagnoses. Patients were well-matched with non-CHD premature infants for GA, BW, and gender, which are all known risk factors for poor outcomes in preterm infants.

The study has limitations. We limited our outcomes to the initial hospital stay. This restricted our ability to examine mortality and other comorbidities beyond discharge from the hospital. The current study did not include the specific details of surgical repair which might be institutionally different across the United States. The impact of this limitation on the provided estimations of outcomes in premature infants might be minimal given most patients with severe CHD do not undergo surgical repair or palliation until they reach a reasonable weight and corrected GA.

## Conclusion

Premature infants with severe CHD are at high risk of neonatal morbidity and mortality. Morbidity remains increased across all GA groups and in all CHD categories. This significant risk of adverse outcomes is important to acknowledge when managing this patient population and when counseling their families. Future research is needed to examine the impact of specific rather than categorized CHD on neonatal outcomes.

## Data Availability

The original contributions presented in the study are included in the article/**Supplementary Material**, further inquiries can be directed to the corresponding author.

## Supplementary material

SUPPLEMENTARY FIGURE S1

Frequency of different categories of CHD in premature infants based on gestational age in weeks.

SUPPLEMENTARY TABLE S1

List of CHDs included in the study with the associated ICD-10 codes.

## References

[1] Liu L Oza S Hogan D Perin J Rudan I Lawn JE Global, regional, and national causes of child mortality in 2000–13, with projections to inform post-2015 priorities: an updated systematic analysis. Lancet. (2015) 385:430–40. 10.1016/S0140-6736(14)61698-6 25280870

[2] Helenius K Sjörs G Shah PS Modi N Reichman B Morisaki N International network for evaluating outcomes (iNeo) of neonates. Survival in very preterm infants: an international comparison of 10 national neonatal networks. Pediatrics. (2017) 140(6):e20171264. 10.1542/peds.2017-1264 29162660

[3] Norman M Håkansson S Kusuda S Vento M Lehtonen L Reichman B International network for evaluation of outcomes in neonates (iNeo) investigators* †. neonatal outcomes in very preterm infants with severe congenital heart defects: an international cohort study. J Am Heart Assoc. (2020) 9(5):e015369. 10.1161/JAHA.119.015369 32079479 PMC7335543

[4] Hoffman JIE Kaplan S. The incidence of congenital heart disease. J Am Coll Cardiol. (2002) 39:1890–900. 10.1016/S0735-1097(02)01886-7 12084585

[5] Reller MD Strickland MJ Riehle-Colarusso T Mahle WT Correa A. Prevalence of congenital heart defects in metropolitan Atlanta, 1998–2005. J Pediatr. (2008) 153:807–13. 10.1016/j.jpeds.2008.05.059 18657826 PMC2613036

[6] Tanner K Sabrine N Wren C. Cardiovascular malformations among preterm infants. Pediatrics. (2005) 16:e833–8. 10.1542/peds.2005-0397 16322141

[7] Matthiesen NB Østergaard JR Hjortdal VE Henriksen TB. Congenital heart defects and the risk of spontaneous preterm birth. J Pediatr. (2021) 229:168–74.e5. 10.1016/j.jpeds.2020.09.059 32980375

[8] Laas E Lelong N Thieulin AC Houyel L Bonnet D Ancel PY Preterm birth and congenital heart defects: a population-based study. Pediatrics. (2012) 130:e829–37. 10.1542/peds.2011-3279 22945415

[9] Dumitrascu Biris I Mintoft A Harris C Rawn Z Jheeta JS Pushparajah K Mortality and morbidity in preterm infants with congenital heart disease. Acta Paediatr. (2022) 111(1):151–6. 10.1111/apa.16155 34655490

[10] Anderson AW Smith PB Corey KM Hill KD Zimmerman KO Clark RH Clinical outcomes in very low BW infants with major congenital heart defects. Early Hum Dev. (2014) 90:791–5. 10.1016/j.earlhumdev.2014.09.006 25463822 PMC4312193

[11] Archer JM Yeager SB Kenny MJ Soll RF Horbar JD. Distribution of and mortality from serious congenital heart disease in very low BW infants. Pediatrics. (2011) 127:293–9. 10.1542/peds.2010-0418 21220403

[12] Agency for Healthcare Research and Quality. Overview of the National (Nationwide) Inpatient Sample (NIS). Available online at: https://www.hcup-us.ahrq.gov/nisoverview.jsp (Accessed February 15, 2024).

[13] Steurer MA Baer RJ Keller RL Oltman S Chambers CD Norton ME Gestational age and outcomes in critical congenital heart disease. Pediatrics. (2017) 140(4):e20170999. 10.1542/peds.2017-0999 28885171

[14] Nembhard WN Salemi JL Hauser KW Kornosky JL. Are there ethnic disparities in risk of preterm birth among infants born with congenital heart defects? Birth Defects Res A Clin Mol Teratol. (2007) 79:754–64. 10.1002/bdra.20411 17990335

[15] Desai J Aggarwal S Lipshultz S Agarwal P Yigazu P Patel R Surgical interventions in infants born preterm with congenital heart defects: an analysis of the Kids’ inpatient database. J Pediatr. (2017) 191:103–9.e4. 10.1016/j.jpeds.2017.07.015 28964428

[16] Nelson JS Strassle PD. Regional differences in right versus left congenital heart disease diagnoses in neonates in the United States. Birth Defects Res. (2018) 110(4):325–35. 10.1002/bdr2.1140 29106052

[17] Peyvandi S Baer RJ Chambers CD Norton ME Rajagopal S Ryckman KK Environmental and socioeconomic factors influence the live-born incidence of congenital heart disease: a population-based study in California. J Am Heart Assoc. (2020) 9(8):e015255. 10.1161/JAHA.119.015255 32306820 PMC7428546

[18] Enders C Pearson D Harley K Ebisu K. Exposure to coarse particulate matter during gestation and term low birthweight in California: variation in exposure and risk across region and socioeconomic subgroup. Sci Total Environ. (2019) 653:1435–44. 10.1016/j.scitotenv.2018.10.323 30759582

[19] GBD 2017 Congenital Heart Disease Collaborators. Global, regional, and national burden of congenital heart disease, 1990–2017: a systematic analysis for the global burden of disease study 2017. Lancet Child Adolesc Health. (2020) 4(3):185–200. 10.1016/S2352-4642(19)30402-X 31978374 PMC7645774

[20] Oster ME Lee KA Honein MA Riehle-Colarusso T Shin M Correa A. Temporal trends in survival among infants with critical congenital heart defects. Pediatrics. (2013) 131:e1502–8. 10.1542/peds.2012-3435 23610203 PMC4471949

[21] Polito A Piga S Cogo PE Corchia C Carnielli V Da Fre M Increased morbidity and mortality in very preterm/VLBW infants with congenital heart disease. Intensive Care Med. (2013) 39:1104–12. 10.1007/s00134-013-2887-y 23536167

[22] Best KE Tennant PWG Rankin J. Survival, by BW and gestational age, in individuals with congenital heart disease: a population-based study. J Am Heart Assoc. (2017) 6(7):e005213. 10.1161/JAHA.116.005213 28733436 PMC5586271

[23] Giannone PJ Luce WA Nankervis CA Hoffman TM Wold LE. Necrotizing enterocolitis in neonates with congenital heart disease. Life Sci. (2008) 82(7–8):341–7. 10.1016/j.lfs.2007.09.036 18187159

[24] McElhinney DB Hedrick HL Bush DM Pereira GR Stafford PW Gaynor JW Necrotizing enterocolitis in neonates with congenital heart disease: risk factors and outcomes. Pediatrics. (2000) 106(5):1080–7. 10.1542/peds.106.5.1080 11061778

[25] Kelleher ST McMahon CJ James A. Necrotizing enterocolitis in children with congenital heart disease: a literature review. Pediatr Cardiol. (2021) 42(8):1688–99. 10.1007/s00246-021-02691-1 34510235 PMC8557173

[26] Carlo WF Kimball TR Michelfelder EC Border WL. Persistent diastolic flow reversal in abdominal aortic Doppler-flow profiles is associated with an increased risk of necrotizing enterocolitis in term infants with congenital heart disease. Pediatrics. (2007) 119(2):330–5. 10.1542/peds.2006-2640 17272623

[27] Cheung PY Hajihosseini M Dinu IA Switzer H Joffe AR Bond GY Outcomes of preterm infants with congenital heart defects after early surgery: defining risk factors at different time points during hospitalization. Front Pediatr. (2021) 28(8):616659. 10.3389/fped.2020.616659 PMC7876369 33585367

[28] Bain J Benjamin DK Jr Hornik CP Benjamin DK Clark R Smith PB. Risk of necrotizing enterocolitis in very-low-birth-weight infants with isolated atrial and ventricular septal defects. J Perinatol. (2014) 34:319–21. 10.1038/jp.2013.174 24434778 PMC3969778

[29] Stoll BJ Hansen NI Bell EF Shankaran S Laptook AR Walsh MC Neonatal outcomes of extremely preterm infants from the NICHD neonatal research network. Pediatrics. (2010) 126(3):443–56. 10.1542/peds.2009-2959 20732945 PMC2982806

[30] Costello JM Polito A Brown DW McElrath TF Graham DA Thiagarajan RR Birth before 39 weeks’ gestation is associated with worse outcomes in neonates with heart disease. Pediatrics. (2010) 26(2):277–84. 10.1542/peds.2009-3640 20603261

[31] Pappas A Shankaran S Hansen NI Bell EF Stoll BJ Laptook AR Outcome of extremely preterm infants (<1,000 g) with congenital heart defects from the national institute of child health and human development neonatal research network. Pediatr Cardiol. (2012) 33(8):1415–26. 10.1007/s00246-012-0375-8 22644414 PMC3687358

[32] Ortinau CM Anadkat JS Smyser CD Eghtesady P. Intraventricular hemorrhage in moderate to severe congenital heart disease. Pediatr Crit Care Med. (2018) 19(1):56–63. 10.1097/PCC.0000000000001374 29210924 PMC5777323

[33] Katz JA Levy PT Butler SC Sadhwani A Lakshminrusimha S Morton SU Preterm congenital heart disease and neurodevelopment: the importance of looking beyond the initial hospitalization. J Perinatol. (2023) 43(7):958–62. 10.1038/s41372-023-01687-4 37179381

[34] Levy PT Thomas AR Wethall A Perez D Steurer M Ball MK. Rethinking congenital heart disease in preterm neonates. Neoreviews. (2022) 23(6):e373–87. 10.1542/neo.23-6-e373 35641458

[35] Gaynor JW Stopp C Wypij D Andropoulos DB Atallah J Atz AM Neurodevelopmental outcomes after cardiac surgery in infancy. Pediatrics. (2015) 135(5):816–25. 10.1542/peds.2014-3825 25917996 PMC4533222

[36] Kumar KR Clark DA Kim EM Perry JD Wright K Thomas SA Association of atrial septal defects and bronchopulmonary dysplasia in premature infants. J Pediatr. (2018) 202:56–62.e2. 10.1016/j.jpeds.2018.07.024 30172431 PMC6317846

