# Supplementary figures and images for: Neonatal outcomes in preterm infants with severe congenital heart disease: a national cohort analysis
Source: Front Pediatr. 2024 Apr 25;12:1326804. doi: 10.3389/fped.2024.1326804 (PMC11079131; doi:10.3389/fped.2024.1326804)

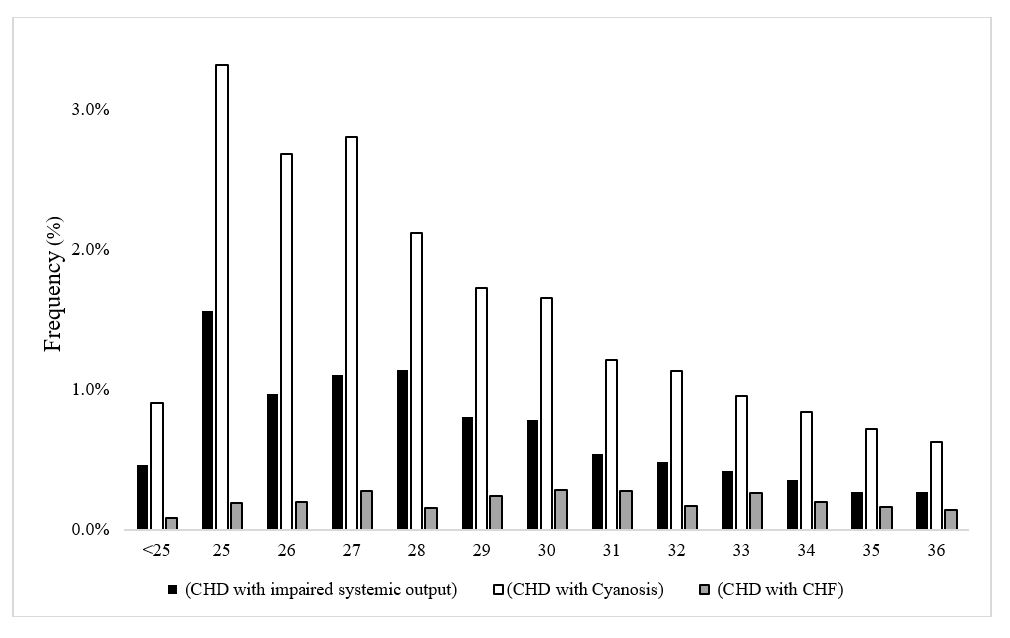

Supplement: Supplementary file 1 [file Image1.jpeg]
