# Supplementary material for: Neonatal outcomes in preterm infants with severe congenital heart disease: a national cohort analysis
Source: Front Pediatr. 2024 Apr 25;12:1326804. doi: 10.3389/fped.2024.1326804 (PMC11079131; doi:10.3389/fped.2024.1326804)
Supplement: Supplementary file 2 [file Table1.pdf]

| Congenital Heart Disease | ICD-10 Code |
|---|---|
| **Category A: Left-sided lesions** | |
| - Aortic valve stenosis | Q23.0 |
| - Aortic valve insufficiency | Q23.1 |
| - Congenital mitral stenosis | Q23.2 |
| - Congenital mitral insufficiency | Q23.3 |
| - Coarctation of the aorta | Q25.1 |
| - Interrupted aortic arch | Q25.21 |
| - Hypoplastic left heart syndrome | Q23.4 |
| - Subvalvar and Supravalvar Aortic stenosis | Q24.4 |
| - Supravalvar Aortic stenosis | Q25.3 |
| **Category B: Cyanotic CHD** | |
| - Tetralogy of Fallot | Q21.3 |
| - Transposition of the great arteries | Q20.3 |
| - Pulmonary valve atresia | Q22.0, Q25.5 |
| - Ebstein anomaly | Q22.5 |
| - Tricuspid atresia | Q22.4 |
| - Total anomalous pulmonary venous return | Q26.2 |
| - Hypoplastic right heart syndrome (other) | Q22.6, Q22.8, Q22.9 |
| **Category C: Shunt lesions** | |
| - Atrioventricular septal defects | Q21.2, Q21.20, Q21.21, Q21.22, Q21.23 |
| - Aortopulmonary septal defect | Q21.4 |
| - Double Outlet right ventricles | Q20.1 |
| - Common arterial trunk | Q20.0 |
| - Aortopulmonary septal defect | Q21.4 |
| - Double outlet right ventricle | Q20.1 |
